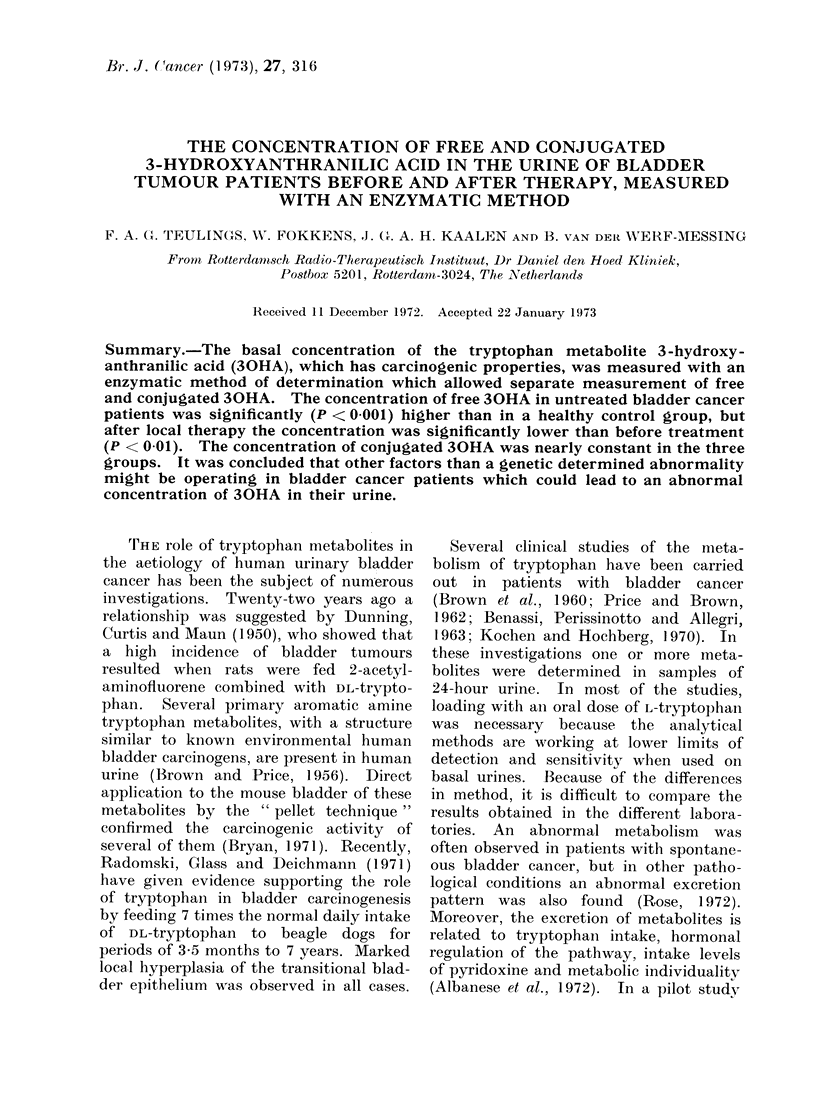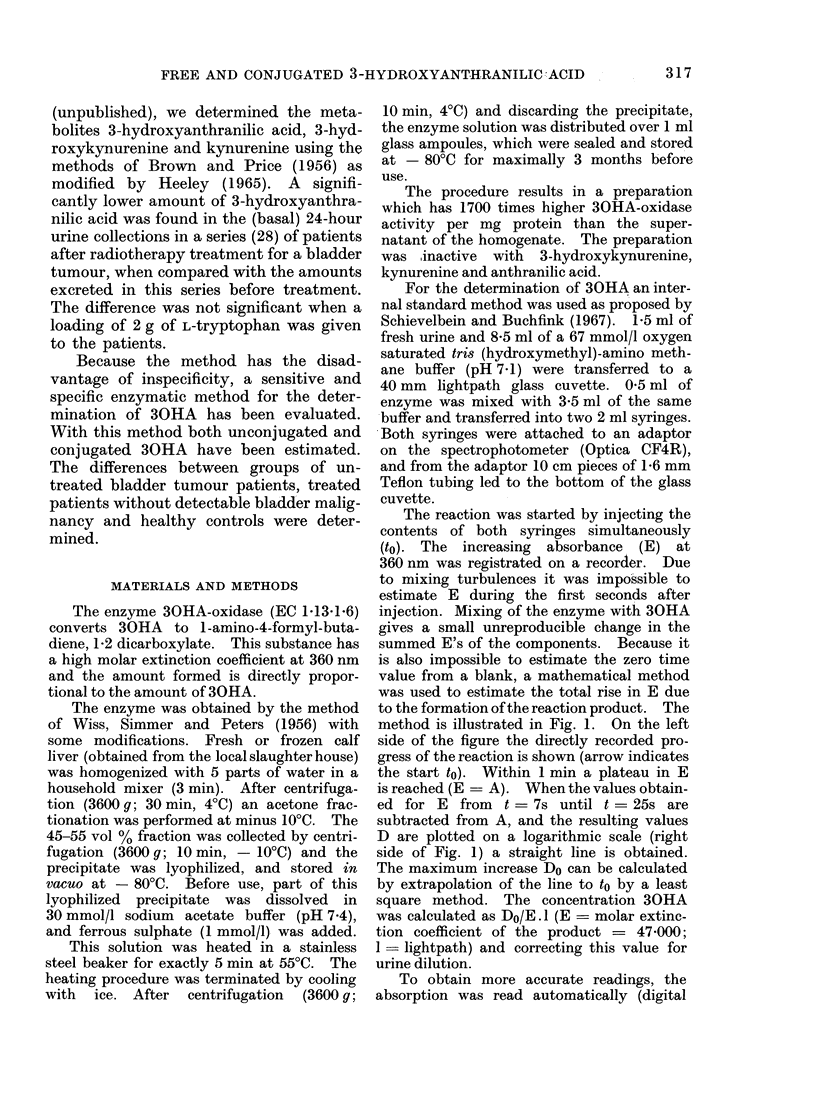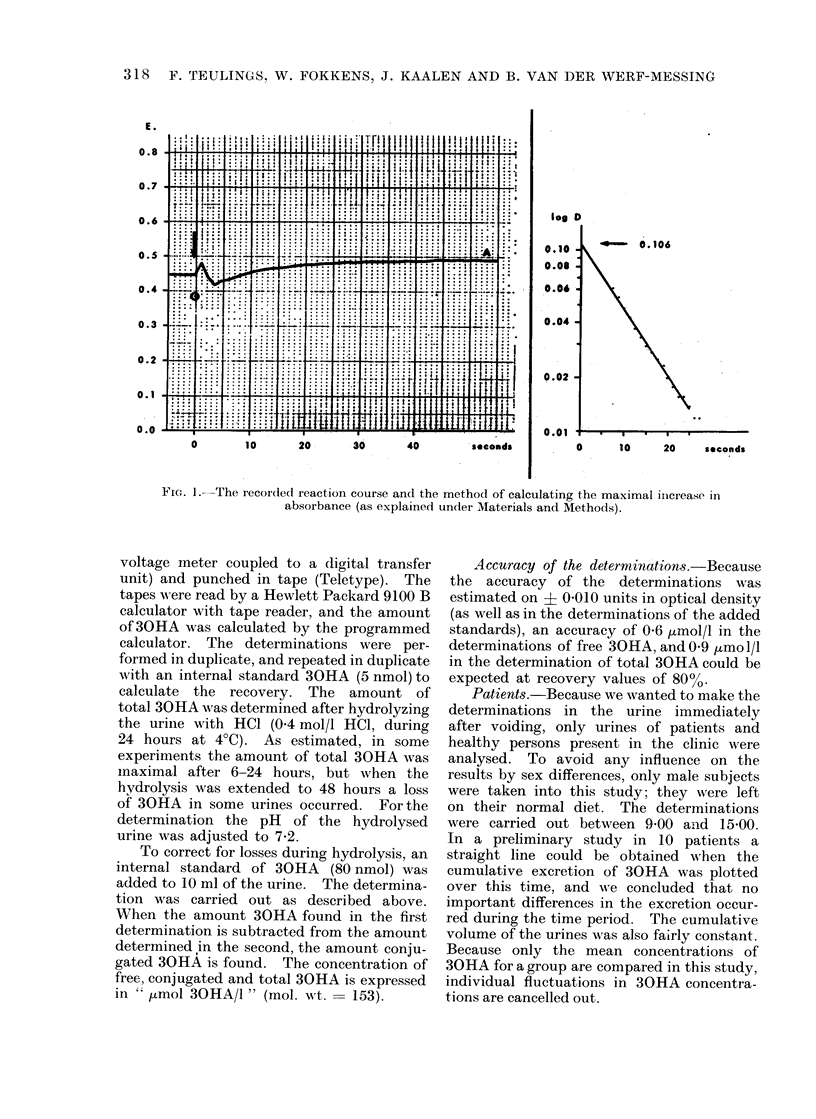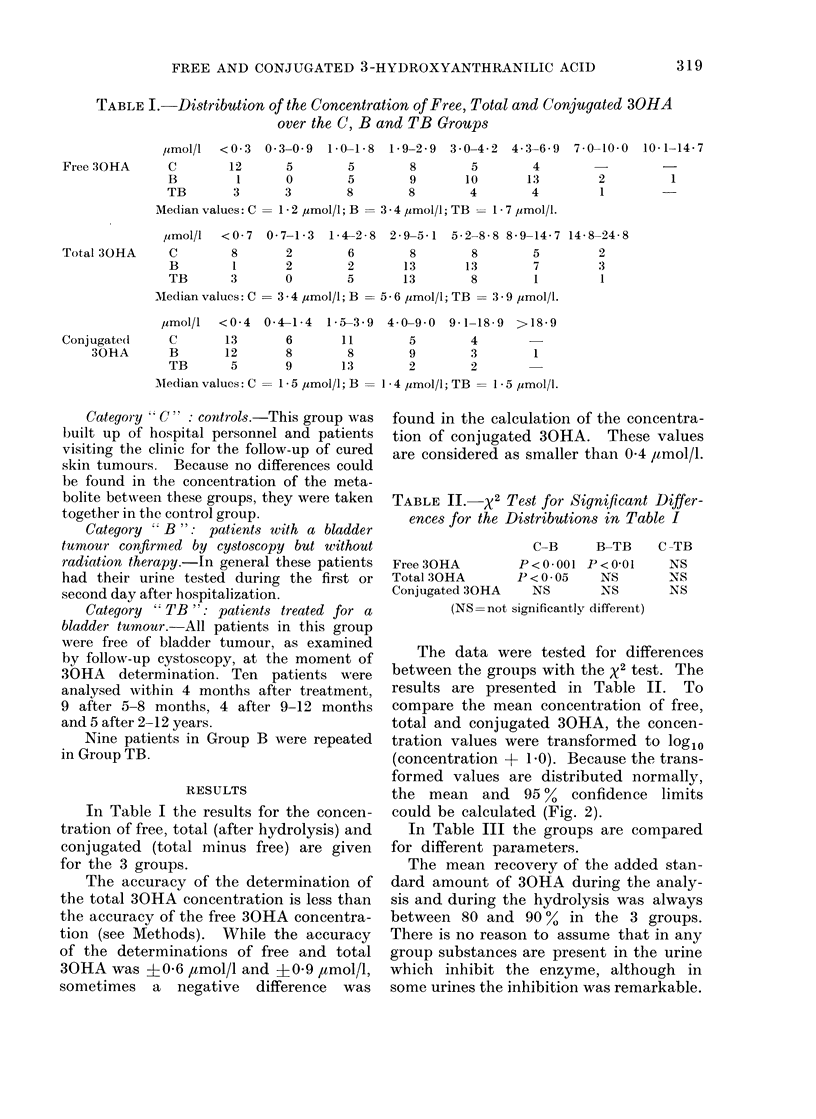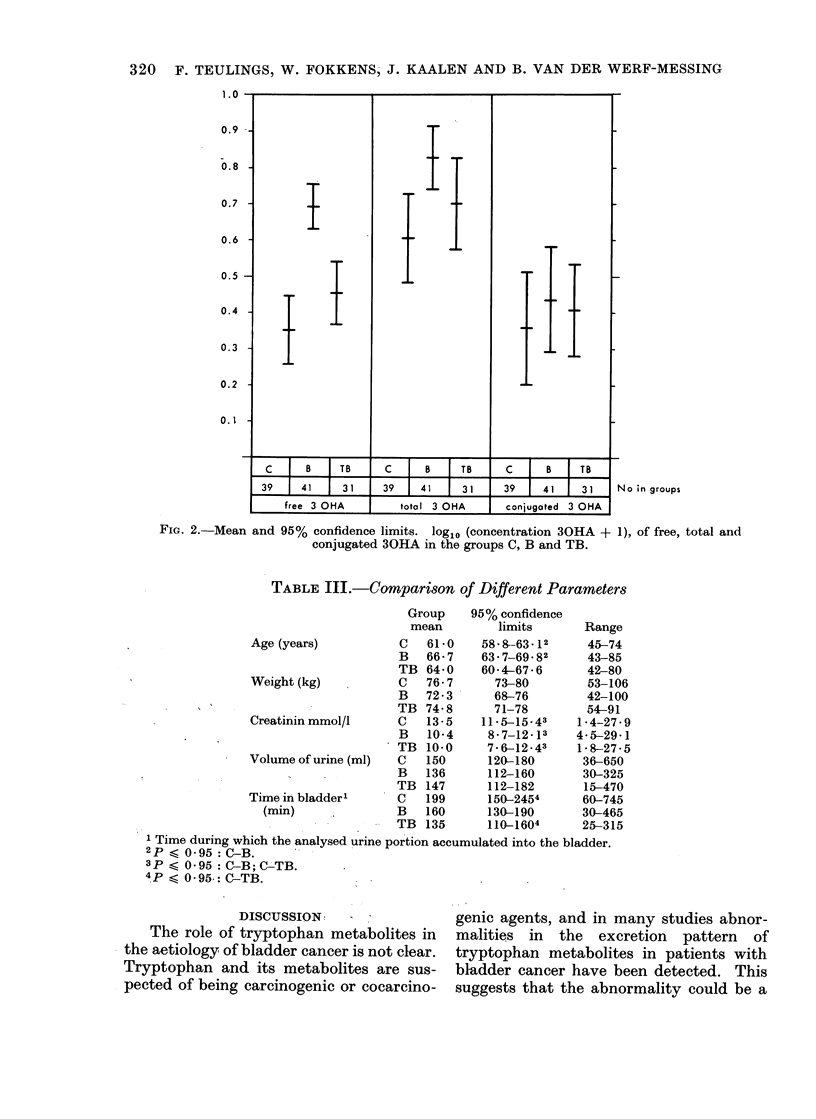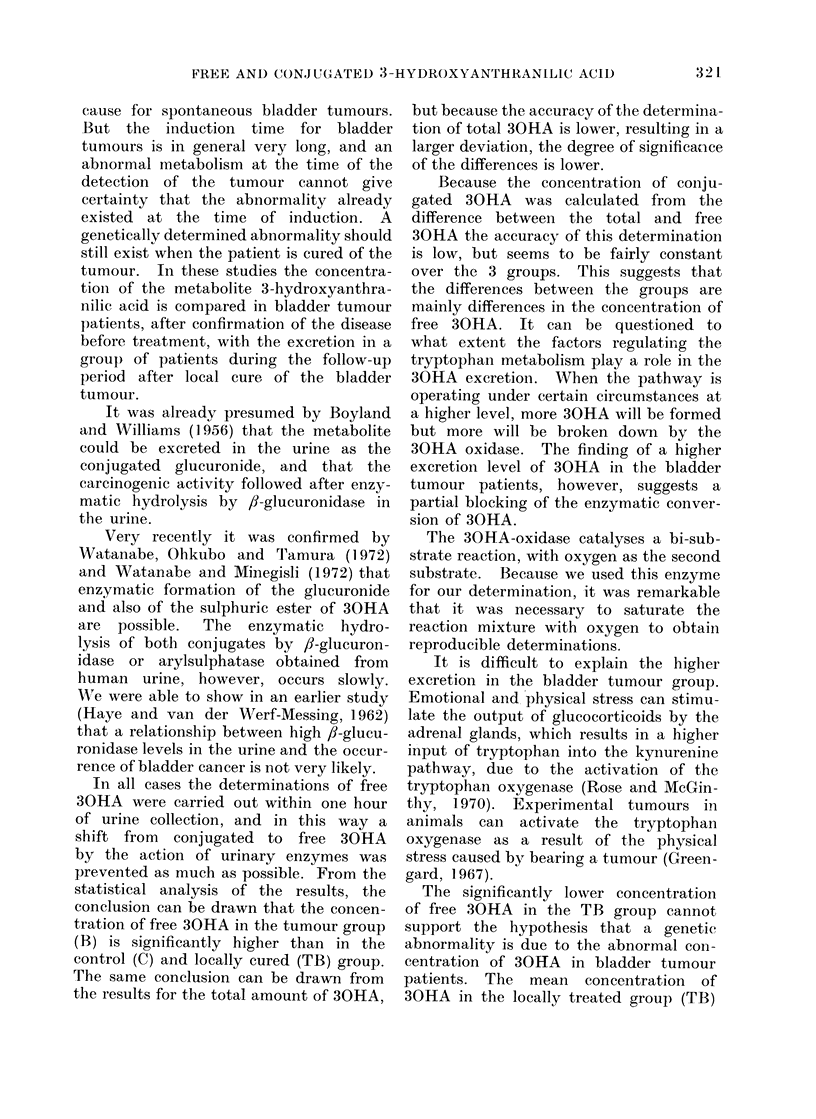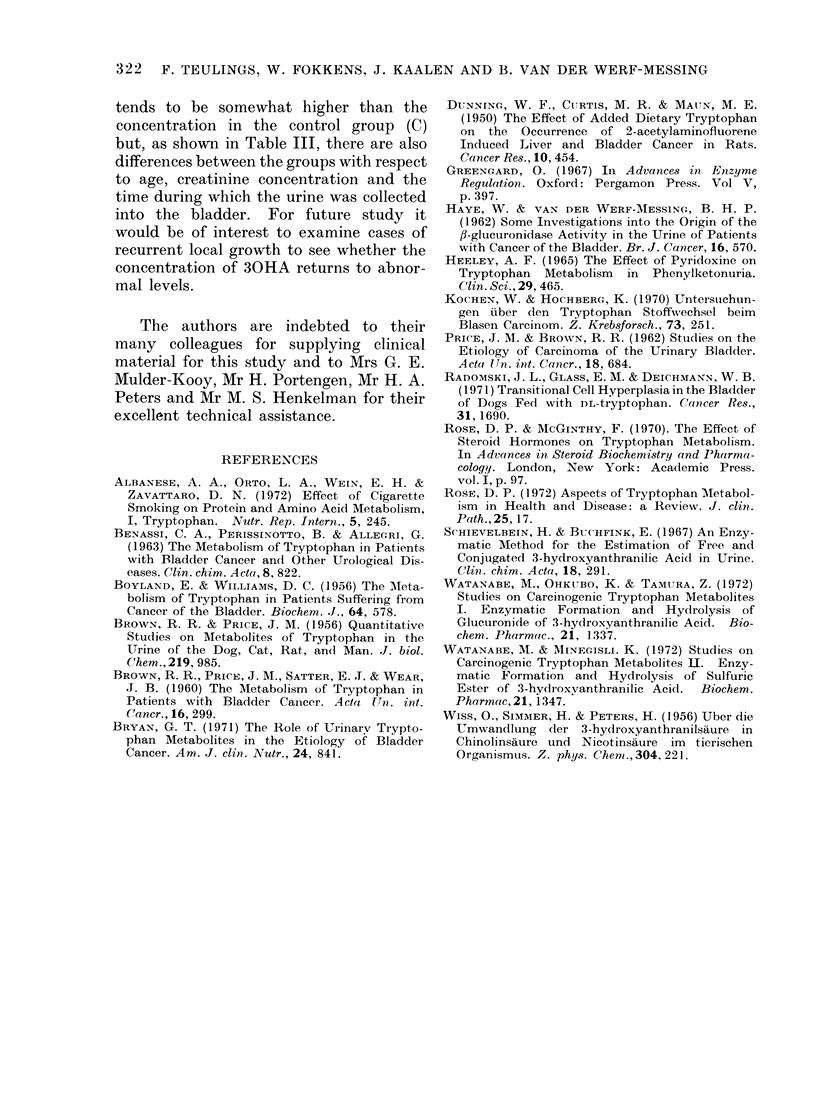# The Concentration of Free and Conjugated 3-Hydroxyanthranilic Acid in the Urine of Bladder Tumour Patients Before and After Therapy, Measured with an Enzymatic Method

**DOI:** 10.1038/bjc.1973.38

**Published:** 1973-04

**Authors:** F. A. G. Teulings, W. Fokkens, J. G. A. H. Kaalen, B. van der Werf-Messing

## Abstract

The basal concentration of the tryptophan metabolite 3-hydroxyanthranilic acid (3OHA), which has carcinogenic properties, was measured with an enzymatic method of determination which allowed separate measurement of free and conjugated 3OHA. The concentration of free 3OHA in untreated bladder cancer patients was significantly (*P* <0·001) higher than in a healthy control group, but after local therapy the concentration was significantly lower than before treatment (*P* <0·01). The concentration of conjugated 3OHA was nearly constant in the three groups. It was concluded that other factors than a genetic determined abnormality might be operating in bladder cancer patients which could lead to an abnormal concentration of 3OHA in their urine.


Br. J. Cancer (1973), 27, 316

### THE CONCENTRATION OF FREE AND CONJUGATED 3-HYDROXYANTHRANILIC ACID IN THE URINE OF BLADDER TUMOUR PATIENTS BEFORE AND AFTER THERAPY, MEASURED WITH AN ENZYMATIC METHOD

F. A. G. TEULINGS, W. FOKKENS, J. G. A. H. KAALEN AND B. VAN DER WERF-MESSING

*From Rotterdamsch Radio-Therapeutisch Instituut, Dr Daniel den Hoed Kliniek, Postbox 5201, Rotterdam-3024, The Netherlands*

Received 11 December 1972. Accepted 22 January 1973

**Summary.—The basal concentration of the tryptophan metabolite 3-hydroxyanthranilic acid (3OHA), which has carcinogenic properties, was measured with an enzymatic method of determination which allowed separate measurement of free and conjugated 3OHA. The concentration of free 3OHA in untreated bladder cancer patients was significantly ($P < 0{\cdot}001$) higher than in a healthy control group, but after local therapy the concentration was significantly lower than before treatment ($P < 0{\cdot}01$). The concentration of conjugated 3OHA was nearly constant in the three groups. It was concluded that other factors than a genetic determined abnormality might be operating in bladder cancer patients which could lead to an abnormal concentration of 3OHA in their urine.**

THE role of tryptophan metabolites in the aetiology of human urinary bladder cancer has been the subject of numerous investigations. Twenty-two years ago a relationship was suggested by Dunning, Curtis and Maun (1950), who showed that a high incidence of bladder tumours resulted when rats were fed 2-acetyl-aminofluorene combined with DL-tryptophan. Several primary aromatic amine tryptophan metabolites, with a structure similar to known environmental human bladder carcinogens, are present in human urine (Brown and Price, 1956). Direct application to the mouse bladder of these metabolites by the " pellet technique " confirmed the carcinogenic activity of several of them (Bryan, 1971). Recently, Radomski, Glass and Deichmann (1971) have given evidence supporting the role of tryptophan in bladder carcinogenesis by feeding 7 times the normal daily intake of DL-tryptophan to beagle dogs for periods of 3·5 months to 7 years. Marked local hyperplasia of the transitional bladder epithelium was observed in all cases.

Several clinical studies of the metabolism of tryptophan have been carried out in patients with bladder cancer (Brown *et al.*, 1960; Price and Brown, 1962; Benassi, Perissinotto and Allegri, 1963; Kochen and Hochberg, 1970). In these investigations one or more metabolites were determined in samples of 24-hour urine. In most of the studies, loading with an oral dose of L-tryptophan was necessary because the analytical methods are working at lower limits of detection and sensitivity when used on basal urines. Because of the differences in method, it is difficult to compare the results obtained in the different laboratories. An abnormal metabolism was often observed in patients with spontaneous bladder cancer, but in other pathological conditions an abnormal excretion pattern was also found (Rose, 1972). Moreover, the excretion of metabolites is related to tryptophan intake, hormonal regulation of the pathway, intake levels of pyridoxine and metabolic individuality (Albanese *et al.*, 1972). In a pilot study

(unpublished), we determined the metabolites 3-hydroxyanthranilic acid, 3-hydroxykynurenine and kynurenine using the methods of Brown and Price (1956) as modified by Heeley (1965). A significantly lower amount of 3-hydroxyanthranilic acid was found in the (basal) 24-hour urine collections in a series (28) of patients after radiotherapy treatment for a bladder tumour, when compared with the amounts excreted in this series before treatment. The difference was not significant when a loading of 2 g of L-tryptophan was given to the patients.

Because the method has the disadvantage of inspecificity, a sensitive and specific enzymatic method for the determination of 3OHA has been evaluated. With this method both unconjugated and conjugated 3OHA have been estimated. The differences between groups of untreated bladder tumour patients, treated patients without detectable bladder malignancy and healthy controls were determined.

### MATERIALS AND METHODS

The enzyme 3OHA-oxidase (EC 1·13·1·6) converts 3OHA to 1-amino-4-formyl-butadiene, 1·2 dicarboxylate. This substance has a high molar extinction coefficient at 360 nm and the amount formed is directly proportional to the amount of 3OHA.

The enzyme was obtained by the method of Wiss, Simmer and Peters (1956) with some modifications. Fresh or frozen calf liver (obtained from the local slaughter house) was homogenized with 5 parts of water in a household mixer (3 min). After centrifugation (3600 *g*; 30 min, 4°C) an acetone fractionation was performed at minus 10°C. The 45–55 vol % fraction was collected by centrifugation (3600 *g*; 10 min, − 10°C) and the precipitate was lyophilized, and stored *in vacuo* at − 80°C. Before use, part of this lyophilized precipitate was dissolved in 30 mmol/l sodium acetate buffer (pH 7·4), and ferrous sulphate (1 mmol/l) was added.

This solution was heated in a stainless steel beaker for exactly 5 min at 55°C. The heating procedure was terminated by cooling with ice. After centrifugation (3600 *g*; 10 min, 4°C) and discarding the precipitate, the enzyme solution was distributed over 1 ml glass ampoules, which were sealed and stored at − 80°C for maximally 3 months before use.

The procedure results in a preparation which has 1700 times higher 3OHA-oxidase activity per mg protein than the supernatant of the homogenate. The preparation was inactive with 3-hydroxykynurenine, kynurenine and anthranilic acid.

For the determination of 3OHA an internal standard method was used as proposed by Schievelbein and Buchfink (1967). 1·5 ml of fresh urine and 8·5 ml of a 67 mmol/l oxygen saturated *tris* (hydroxymethyl)-amino methane buffer (pH 7·1) were transferred to a 40 mm lightpath glass cuvette. 0·5 ml of enzyme was mixed with 3·5 ml of the same buffer and transferred into two 2 ml syringes. Both syringes were attached to an adaptor on the spectrophotometer (Optica CF4R), and from the adaptor 10 cm pieces of 1·6 mm Teflon tubing led to the bottom of the glass cuvette.

The reaction was started by injecting the contents of both syringes simultaneously ($t_0$). The increasing absorbance (E) at 360 nm was registrated on a recorder. Due to mixing turbulences it was impossible to estimate E during the first seconds after injection. Mixing of the enzyme with 3OHA gives a small unreproducible change in the summed E's of the components. Because it is also impossible to estimate the zero time value from a blank, a mathematical method was used to estimate the total rise in E due to the formation of the reaction product. The method is illustrated in Fig. 1. On the left side of the figure the directly recorded progress of the reaction is shown (arrow indicates the start $t_0$). Within 1 min a plateau in E is reached (E = A). When the values obtained for E from $t = 7$s until $t = 25$s are subtracted from A, and the resulting values D are plotted on a logarithmic scale (right side of Fig. 1) a straight line is obtained. The maximum increase $D_0$ can be calculated by extrapolation of the line to $t_0$ by a least square method. The concentration 3OHA was calculated as $D_0/E.l$ (E = molar extinction coefficient of the product = 47·000; l = lightpath) and correcting this value for urine dilution.

To obtain more accurate readings, the absorption was read automatically (digital

FIG. 1.—The recorded reaction course and the method of calculating the maximal increase in absorbance (as explained under Materials and Methods).

voltage meter coupled to a digital transfer unit) and punched in tape (Teletype). The tapes were read by a Hewlett Packard 9100 B calculator with tape reader, and the amount of 3OHA was calculated by the programmed calculator. The determinations were performed in duplicate, and repeated in duplicate with an internal standard 3OHA (5 nmol) to calculate the recovery. The amount of total 3OHA was determined after hydrolyzing the urine with HCl (0·4 mol/l HCl, during 24 hours at 4°C). As estimated, in some experiments the amount of total 3OHA was maximal after 6–24 hours, but when the hydrolysis was extended to 48 hours a loss of 3OHA in some urines occurred. For the determination the pH of the hydrolysed urine was adjusted to 7·2.

To correct for losses during hydrolysis, an internal standard of 3OHA (80 nmol) was added to 10 ml of the urine. The determination was carried out as described above. When the amount 3OHA found in the first determination is subtracted from the amount determined in the second, the amount conjugated 3OHA is found. The concentration of free, conjugated and total 3OHA is expressed in " $\mu$mol 3OHA/l " (mol. wt. = 153).

*Accuracy of the determinations.*—Because the accuracy of the determinations was estimated on $\pm$ 0·010 units in optical density (as well as in the determinations of the added standards), an accuracy of 0·6 $\mu$mol/l in the determinations of free 3OHA, and 0·9 $\mu$mol/l in the determination of total 3OHA could be expected at recovery values of 80%.

*Patients.*—Because we wanted to make the determinations in the urine immediately after voiding, only urines of patients and healthy persons present in the clinic were analysed. To avoid any influence on the results by sex differences, only male subjects were taken into this study; they were left on their normal diet. The determinations were carried out between 9·00 and 15·00. In a preliminary study in 10 patients a straight line could be obtained when the cumulative excretion of 3OHA was plotted over this time, and we concluded that no important differences in the excretion occurred during the time period. The cumulative volume of the urines was also fairly constant. Because only the mean concentrations of 3OHA for a group are compared in this study, individual fluctuations in 3OHA concentrations are cancelled out.

TABLE I.—*Distribution of the Concentration of Free, Total and Conjugated 3OHA over the C, B and TB Groups*

| | μmol/l | <0·3 | 0·3–0·9 | 1·0–1·8 | 1·9–2·9 | 3·0–4·2 | 4·3–6·9 | 7·0–10·0 | 10·1–14·7 |
|---|---|---|---|---|---|---|---|---|---|
| Free 3OHA | C | 12 | 5 | 5 | 8 | 5 | 4 | — | — |
| | B | 1 | 0 | 5 | 9 | 10 | 13 | 2 | 1 |
| | TB | 3 | 3 | 8 | 8 | 4 | 4 | 1 | — |

Median values: C = 1·2 μmol/l; B = 3·4 μmol/l; TB = 1·7 μmol/l.

| | μmol/l | <0·7 | 0·7–1·3 | 1·4–2·8 | 2·9–5·1 | 5·2–8·8 | 8·9–14·7 | 14·8–24·8 |
|---|---|---|---|---|---|---|---|---|
| Total 3OHA | C | 8 | 2 | 6 | 8 | 8 | 5 | 2 |
| | B | 1 | 2 | 2 | 13 | 13 | 7 | 3 |
| | TB | 3 | 0 | 5 | 13 | 8 | 1 | 1 |

Median values: C = 3·4 μmol/l; B = 5·6 μmol/l; TB = 3·9 μmol/l.

| | μmol/l | <0·4 | 0·4–1·4 | 1·5–3·9 | 4·0–9·0 | 9·1–18·9 | >18·9 |
|---|---|---|---|---|---|---|---|
| Conjugated 3OHA | C | 13 | 6 | 11 | 5 | 4 | — |
| | B | 12 | 8 | 8 | 9 | 3 | 1 |
| | TB | 5 | 9 | 13 | 2 | 2 | — |

Median values: C = 1·5 μmol/l; B = 1·4 μmol/l; TB = 1·5 μmol/l.

*Category "C" : controls.*—This group was built up of hospital personnel and patients visiting the clinic for the follow-up of cured skin tumours. Because no differences could be found in the concentration of the metabolite between these groups, they were taken together in the control group.

*Category "B": patients with a bladder tumour confirmed by cystoscopy but without radiation therapy.*—In general these patients had their urine tested during the first or second day after hospitalization.

*Category "TB": patients treated for a bladder tumour.*—All patients in this group were free of bladder tumour, as examined by follow-up cystoscopy, at the moment of 3OHA determination. Ten patients were analysed within 4 months after treatment, 9 after 5–8 months, 4 after 9–12 months and 5 after 2–12 years.

Nine patients in Group B were repeated in Group TB.

### RESULTS

In Table I the results for the concentration of free, total (after hydrolysis) and conjugated (total minus free) are given for the 3 groups.

The accuracy of the determination of the total 3OHA concentration is less than the accuracy of the free 3OHA concentration (see Methods). While the accuracy of the determinations of free and total 3OHA was ±0·6 μmol/l and ±0·9 μmol/l, sometimes a negative difference was found in the calculation of the concentration of conjugated 3OHA. These values are considered as smaller than 0·4 μmol/l.

TABLE II.—$\chi^2$ *Test for Significant Differences for the Distributions in Table I*

| | C–B | B–TB | C–TB |
|---|---|---|---|
| Free 3OHA | $P<0·001$ | $P<0·01$ | NS |
| Total 3OHA | $P<0·05$ | NS | NS |
| Conjugated 3OHA | NS | NS | NS |

(NS = not significantly different)

The data were tested for differences between the groups with the $\chi^2$ test. The results are presented in Table II. To compare the mean concentration of free, total and conjugated 3OHA, the concentration values were transformed to $\log_{10}$ (concentration + 1·0). Because the transformed values are distributed normally, the mean and 95% confidence limits could be calculated (Fig. 2).

In Table III the groups are compared for different parameters.

The mean recovery of the added standard amount of 3OHA during the analysis and during the hydrolysis was always between 80 and 90% in the 3 groups. There is no reason to assume that in any group substances are present in the urine which inhibit the enzyme, although in some urines the inhibition was remarkable.

FIG. 2.—Mean and 95% confidence limits. $\log_{10}$ (concentration 3OHA + 1), of free, total and conjugated 3OHA in the groups C, B and TB.

TABLE III.—*Comparison of Different Parameters*

| | Group | mean | 95% confidence limits | Range |
|---|---|---|---|---|
| Age (years) | C | 61·0 | 58·8–63·1[2] | 45–74 |
| | B | 66·7 | 63·7–69·8[2] | 43–85 |
| | TB | 64·0 | 60·4–67·6 | 42–80 |
| Weight (kg) | C | 76·7 | 73–80 | 53–106 |
| | B | 72·3 | 68–76 | 42–100 |
| | TB | 74·8 | 71–78 | 54–91 |
| Creatinin mmol/l | C | 13·5 | 11·5–15·4[3] | 1·4–27·9 |
| | B | 10·4 | 8·7–12·1[3] | 4·5–29·1 |
| | TB | 10·0 | 7·6–12·4[3] | 1·8–27·5 |
| Volume of urine (ml) | C | 150 | 120–180 | 36–650 |
| | B | 136 | 112–160 | 30–325 |
| | TB | 147 | 112–182 | 15–470 |
| Time in bladder[1] (min) | C | 199 | 150–245[4] | 60–745 |
| | B | 160 | 130–190 | 30–465 |
| | TB | 135 | 110–160[4] | 25–315 |

[1] Time during which the analysed urine portion accumulated into the bladder.
[2] $P \leqslant 0 \cdot 95$ : C–B.
[3] $P \leqslant 0 \cdot 95$ : C–B; C–TB.
[4] $P \leqslant 0 \cdot 95$ : C–TB.

DISCUSSION

The role of tryptophan metabolites in the aetiology of bladder cancer is not clear. Tryptophan and its metabolites are suspected of being carcinogenic or cocarcinogenic agents, and in many studies abnormalities in the excretion pattern of tryptophan metabolites in patients with bladder cancer have been detected. This suggests that the abnormality could be a cause for spontaneous bladder tumours. But the induction time for bladder tumours is in general very long, and an abnormal metabolism at the time of the detection of the tumour cannot give certainty that the abnormality already existed at the time of induction. A genetically determined abnormality should still exist when the patient is cured of the tumour. In these studies the concentration of the metabolite 3-hydroxyanthranilic acid is compared in bladder tumour patients, after confirmation of the disease before treatment, with the excretion in a group of patients during the follow-up period after local cure of the bladder tumour.

It was already presumed by Boyland and Williams (1956) that the metabolite could be excreted in the urine as the conjugated glucuronide, and that the carcinogenic activity followed after enzymatic hydrolysis by $\beta$-glucuronidase in the urine.

Very recently it was confirmed by Watanabe, Ohkubo and Tamura (1972) and Watanabe and Minegisli (1972) that enzymatic formation of the glucuronide and also of the sulphuric ester of 3OHA are possible. The enzymatic hydrolysis of both conjugates by $\beta$-glucuronidase or arylsulphatase obtained from human urine, however, occurs slowly. We were able to show in an earlier study (Haye and van der Werf-Messing, 1962) that a relationship between high $\beta$-glucuronidase levels in the urine and the occurrence of bladder cancer is not very likely.

In all cases the determinations of free 3OHA were carried out within one hour of urine collection, and in this way a shift from conjugated to free 3OHA by the action of urinary enzymes was prevented as much as possible. From the statistical analysis of the results, the conclusion can be drawn that the concentration of free 3OHA in the tumour group (B) is significantly higher than in the control (C) and locally cured (TB) group. The same conclusion can be drawn from the results for the total amount of 3OHA, but because the accuracy of the determination of total 3OHA is lower, resulting in a larger deviation, the degree of significance of the differences is lower.

Because the concentration of conjugated 3OHA was calculated from the difference between the total and free 3OHA the accuracy of this determination is low, but seems to be fairly constant over the 3 groups. This suggests that the differences between the groups are mainly differences in the concentration of free 3OHA. It can be questioned to what extent the factors regulating the tryptophan metabolism play a role in the 3OHA excretion. When the pathway is operating under certain circumstances at a higher level, more 3OHA will be formed but more will be broken down by the 3OHA oxidase. The finding of a higher excretion level of 3OHA in the bladder tumour patients, however, suggests a partial blocking of the enzymatic conversion of 3OHA.

The 3OHA-oxidase catalyses a bi-substrate reaction, with oxygen as the second substrate. Because we used this enzyme for our determination, it was remarkable that it was necessary to saturate the reaction mixture with oxygen to obtain reproducible determinations.

It is difficult to explain the higher excretion in the bladder tumour group. Emotional and physical stress can stimulate the output of glucocorticoids by the adrenal glands, which results in a higher input of tryptophan into the kynurenine pathway, due to the activation of the tryptophan oxygenase (Rose and McGinthy, 1970). Experimental tumours in animals can activate the tryptophan oxygenase as a result of the physical stress caused by bearing a tumour (Greengard, 1967).

The significantly lower concentration of free 3OHA in the TB group cannot support the hypothesis that a genetic abnormality is due to the abnormal concentration of 3OHA in bladder tumour patients. The mean concentration of 3OHA in the locally treated group (TB)

tends to be somewhat higher than the concentration in the control group (C) but, as shown in Table III, there are also differences between the groups with respect to age, creatinine concentration and the time during which the urine was collected into the bladder. For future study it would be of interest to examine cases of recurrent local growth to see whether the concentration of 3OHA returns to abnormal levels.

The authors are indebted to their many colleagues for supplying clinical material for this study and to Mrs G. E. Mulder-Kooy, Mr H. Portengen, Mr H. A. Peters and Mr M. S. Henkelman for their excellent technical assistance.

### REFERENCES

ALBANESE, A. A., ORTO, L. A., WEIN, E. H. & ZAVATTARO, D. N. (1972) Effect of Cigarette Smoking on Protein and Amino Acid Metabolism, I, Tryptophan. *Nutr. Rep. Intern.*, **5**, 245.

BENASSI, C. A., PERISSINOTTO, B. & ALLEGRI, G. (1963) The Metabolism of Tryptophan in Patients with Bladder Cancer and Other Urological Diseases. *Clin. chim. Acta*, **8**, 822.

BOYLAND, E. & WILLIAMS, D. C. (1956) The Metabolism of Tryptophan in Patients Suffering from Cancer of the Bladder. *Biochem. J.*, **64**, 578.

BROWN, R. R. & PRICE, J. M. (1956) Quantitative Studies on Metabolites of Tryptophan in the Urine of the Dog, Cat, Rat, and Man. *J. biol. Chem.*, **219**, 985.

BROWN, R. R., PRICE, J. M., SATTER, E. J. & WEAR, J. B. (1960) The Metabolism of Tryptophan in Patients with Bladder Cancer. *Acta Un. int. Cancr.*, **16**, 299.

BRYAN, G. T. (1971) The Role of Urinary Tryptophan Metabolites in the Etiology of Bladder Cancer. *Am. J. clin. Nutr.*, **24**, 841.

DUNNING, W. F., CURTIS, M. R. & MAUN, M. E. (1950) The Effect of Added Dietary Tryptophan on the Occurrence of 2-acetylaminofluorene Induced Liver and Bladder Cancer in Rats. *Cancer Res.*, **10**, 454.

GREENGARD, O. (1967) In *Advances in Enzyme Regulation*. Oxford: Pergamon Press. Vol V, p. 397.

HAYE, W. & VAN DER WERF-MESSING, B. H. P. (1962) Some Investigations into the Origin of the β-glucuronidase Activity in the Urine of Patients with Cancer of the Bladder. *Br. J. Cancer*, **16**, 570.

HEELEY, A. F. (1965) The Effect of Pyridoxine on Tryptophan Metabolism in Phenylketonuria. *Clin. Sci.*, **29**, 465.

KOCHEN, W. & HOCHBERG, K. (1970) Untersuchungen über den Tryptophan Stoffwechsel beim Blasen Carcinom. *Z. Krebsforsch.*, **73**, 251.

PRICE, J. M. & BROWN, R. R. (1962) Studies on the Etiology of Carcinoma of the Urinary Bladder. *Acta Un. int. Cancr.*, **18**, 684.

RADOMSKI, J. L., GLASS, E. M. & DEICHMANN, W. B. (1971) Transitional Cell Hyperplasia in the Bladder of Dogs Fed with DL-tryptophan. *Cancer Res.*, **31**, 1690.

ROSE, D. P. & MCGINTHY, F. (1970). The Effect of Steroid Hormones on Tryptophan Metabolism. In *Advances in Steroid Biochemistry and Pharmacology*. London, New York: Academic Press. vol. I, p. 97.

ROSE, D. P. (1972) Aspects of Tryptophan Metabolism in Health and Disease: a Review. *J. clin. Path.*, **25**, 17.

SCHIEVELBEIN, H. & BUCHFINK, E. (1967) An Enzymatic Method for the Estimation of Free and Conjugated 3-hydroxyanthranilic Acid in Urine. *Clin. chim. Acta*, **18**, 291.

WATANABE, M., OHKUBO, K. & TAMURA, Z. (1972) Studies on Carcinogenic Tryptophan Metabolites I. Enzymatic Formation and Hydrolysis of Glucuronide of 3-hydroxyanthranilic Acid. *Biochem. Pharmac.*, **21**, 1337.

WATANABE, M. & MINEGISLI. K. (1972) Studies on Carcinogenic Tryptophan Metabolites II. Enzymatic Formation and Hydrolysis of Sulfuric Ester of 3-hydroxyanthranilic Acid. *Biochem. Pharmac*, **21**, 1347.

WISS, O., SIMMER, H. & PETERS, H. (1956) Uber die Umwandlung der 3-hydroxyanthranilsäure in Chinolinsäure und Nicotinsäure im tierischen Organismus. *Z. phys. Chem.*, **304**, 221.